# Supplementary material for: Microbial communities associated with mounds of the Orange-footed scrubfowl Megapodius reinwardt
Source: PeerJ. 2022 Jul 25;10:e13600. doi: 10.7717/peerj.13600 (PMC9332330; doi:10.7717/peerj.13600)
Supplement: Supplemental Information 1 — (± 1 standard deviation) for the bacterial and fungal taxa sampled for each sample category: faeces, mound, deep soil, shallow soil. [file peerj-10-13600-s001.docx]

| Sample Category | Average Shannon value ± 1 standard deviation | | |
| --- | --- | --- | --- |
|  | Bacteria (16S) | Fungi (18S) |  |
| Faeces | 3.78 ± 0.84 | 2.93 ± 0.46 |  |
| Mound | 6.52 ± 0.87 | 4.02 ± 0.96 |  |
| Deep Soil | 6.64 ± 0.12 | 2.36 ± 1.75 |  |
| Shallow Soil | 6.62 ± 0.64 | 4.30 ± 0.74 |  |
