# Supplementary material for: Microbial communities associated with mounds of the Orange-footed scrubfowl Megapodius reinwardt
Source: PeerJ. 2022 Jul 25;10:e13600. doi: 10.7717/peerj.13600 (PMC9332330; doi:10.7717/peerj.13600)
Supplement: Supplemental Information 2 — Pairwise test-comparisons and number of unique permutations for the bacterial and fungal taxa sampled from the sample categories: faeces, mound, deep soil, shallow soil. [file peerj-10-13600-s002.docx]

| Sample Category comparison groups | p-value (unique perms) | |
| --- | --- | --- |
|  | Bacteria (16S) | Fungi (18S) |
| Mound, Shallow Soil | 0.68 (998) | 0.209 (997) |
| Mound, Deep Soil | 0.76 (337) | 0.667 (404) |
| Mound, Faeces | **0.001** (714) | **0.032** (413) |
| Shallow Soil, Deep Soil | 0.94 (398) | **0.036** (417) |
| Shallow Soil, Faeces | **0.001** (776) | **0.005** (402) |
| Deep Soil, Faeces | **0.03** (35) | 0.198 (10) |
