# Supplementary material for: Microbial communities associated with mounds of the Orange-footed scrubfowl Megapodius reinwardt
Source: PeerJ. 2022 Jul 25;10:e13600. doi: 10.7717/peerj.13600 (PMC9332330; doi:10.7717/peerj.13600)
Supplement: Supplemental Information 3 — PERMANOVA analysis testing for differences in mound-associated taxa between locations (A–D). a) Bacteria in mound samples. b) Fungi in mound samples. “df” degrees of freedom, “ECV” square root of estimates of components of variation indicating the size of the effect due to that factor as average % SV dissimilarity. P value is based on >968 unique permutations. “PermDISP” is a permutational distance-based test for homogeneity among the replicates within the locations. [file peerj-10-13600-s003.docx]

1. Mound samples: Bacteria

| Factor PERMANOVA | Pseudo-F (df) | ECV | P value | PermDISP P value |
| --- | --- | --- | --- | --- |
| Location | 3.1 (3) | 43.3 | <0.001 | 0.07 |

1. Mound samples: Fungi

| Factor PERMANOVA | Pseudo-F (df) | ECV | P value | PermDISP P value |
| --- | --- | --- | --- | --- |
| Location | 3.0 (3) | 47.2 | <0.001 | 0.99 |
