# Supplementary material for: Microbial communities associated with mounds of the Orange-footed scrubfowl Megapodius reinwardt
Source: PeerJ. 2022 Jul 25;10:e13600. doi: 10.7717/peerj.13600 (PMC9332330; doi:10.7717/peerj.13600)
Supplement: Supplemental Information 4 — PERMANOVA analysis testing for differences in shallow soil-associated taxa between locations (A–D). a) Bacteria in shallow soil samples. b) Fungi in shallow soil samples. “df” degrees of freedom, “ECV” square root of estimates of components of variation indicating the size of the effect due to that factor as average % SV dissimilarity. P value is based on >956 unique permutations. “PermDISP” is a permutational distance-based test for homogeneity among the replicates within the locations. [file peerj-10-13600-s004.docx]

1. Shallow soil samples: Bacteria

| Factor PERMANOVA | Pseudo-F (df) | ECV | P value | PermDISP P value |
| --- | --- | --- | --- | --- |
| Location | 2.9 (3) | 45.3 | <0.001 | <0.01 |

1. Shallow soil samples: Fungi

| Factor PERMANOVA | Pseudo-F (df) | ECV | P value | PermDISP P value |
| --- | --- | --- | --- | --- |
| Location | 2.1 (3) | 53.1 | <0.001 | <0.001 |
