# Supplementary figures and images for: Microbial communities associated with mounds of the Orange-footed scrubfowl Megapodius reinwardt
Source: PeerJ. 2022 Jul 25;10:e13600. doi: 10.7717/peerj.13600 (PMC9332330; doi:10.7717/peerj.13600)

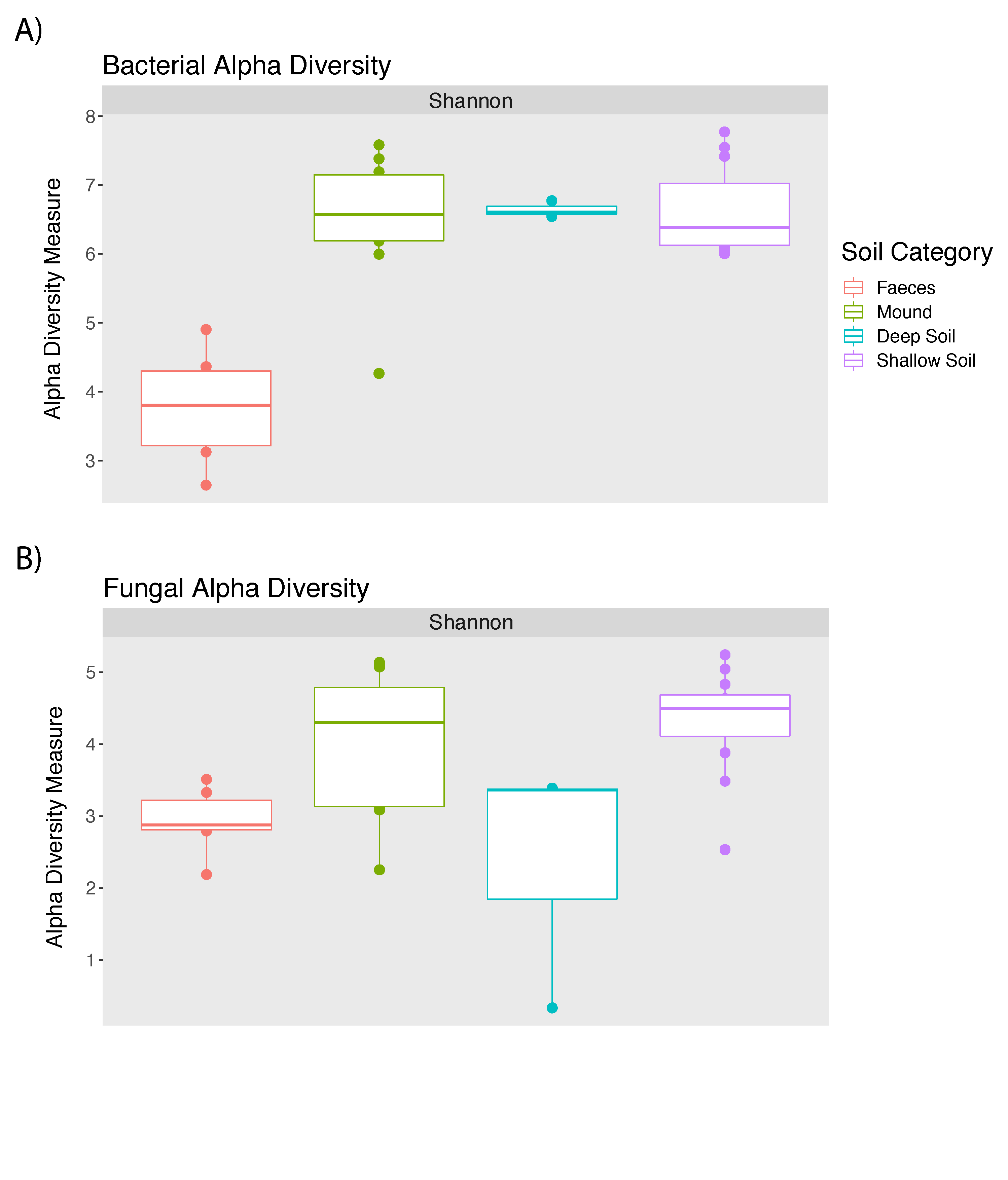

Supplement: Supplemental Information 5 — A) Bacterial alpha diversity. B) Fungal alpha diversity. [file peerj-10-13600-s005.png]
